# From the establishment of a national bioinformatics society to the development of a national bioinformatics infrastructure

**DOI:** 10.12688/f1000research.153895.2

**Published:** 2026-06-17

**Authors:** Bogdan Mirăuță, Cătălina Zenoaga-Barbăroșie, Monica Abrudan, Marius Mihășan, Mădălina Giurgiu, Daria Mihalachi, Michalis-Daniel Lazăr, RSBI Members, Horia L. Banciu

**Affiliations:** 1 Romanian Bioinformatics Cluster, Târgu Mureș, Romania; 2 Romanian Society of Bioinformatics, Cluj-Napoca, Romania; 3 Department of Genetics, Faculty of Biology, University of Bucharest, Bucharest, Romania; 4 Centre for Genomic Pathogen Surveillance, Pandemic Sciences Institute, University of Oxford, Oxford, England, UK; 5 Biology Department, Alexandru Ioan Cuza University of Iași, Iași, Romania; 6 Free University of Berlin, Berlin, Germany; 7 Department of Pediatric Oncology and Hematology, Charité – Universitätsmedizin Berlin, Berlin, Germany; 8 Department of Molecular Biology and Biotechnology, Faculty of Biology and Geology, Babeș-Bolyai University, Cluj-Napoca, Romania

**Keywords:** bioinformatics society, ELIXIR, FAIR, training, public engagement

## Abstract

We describe the evolution of a bioinformatics national capacity from scattered professionals into a collaborative organisation, and advancements in the adoption of the bioinformatics infrastructure philosophy by the national community.

The Romanian Society of Bioinformatics (RSBI), a national professional society, was founded in 2019 to accelerate the development of Romanian bioinformatics. Incrementally, RSBI expanded its role to include: i) developing a community and engaging the public and stakeholders, ii) a national training approach, including through increased interactions with European training resources, and iii) advocating national participation in European bioinformatics infrastructures. In a next step RSBI led the development of the national bioinformatics infrastructure, the Romanian Bioinformatics Cluster (CRB) with the mission to act as an ELIXIR National Node.

In this paper we report both the successful projects in training, public engagement, and policy projects, as well as initiatives related to data federation that, while not successful, can serve as valuable learning experiences for future implementations.

We explain CRB’s structure and the role such an entity can play in the national bioinformatics infrastructure for data, tools, and training. Finally, we offer insights into the evolving role of the bioinformatics professional society and the synergies and interactions with the forthcoming National ELIXIR Node

## Introduction

The need for centralised data resources and bioinformatics services prompted the development of dedicated infrastructures. The establishment in 1994 of the European Bioinformatics Institute (EMBL-EBI), an outstation of European Molecular Biology Laboratory (EMBL) was followed by the development of bioinformatics networks and national bioinformatics infrastructures in most European countries. These infrastructures encompass resources and services that are organised and coordinated at a country level, and aim to provide the necessary tools, resources, and expertise to researchers and organisations within a country. The infrastructures include several key components: collaboration and networking, data, software, computing resources, training, support, ELSI (Ethical, Legal and Societal Implications) and outreach.

In a second phase, the increasing need to have synchronised approaches across Europe led to the development of the distributed infrastructure for life-science data (ELIXIR) ( Crosswell and Thornton, 2012; Harrow et al., 2021). ELIXIR was developed as a project in 2006, established as an intergovernmental research infrastructure in 2013 (the ELIXIR Consortium Agreement) and became an ESFRI (European Strategy Forum on Research and Innovation) landmark in 2016. ELIXIR brings together national centres across Europe into a coordinated infrastructure for bioinformatics.

The relevance of such organisations became obvious during the COVID-19 pandemic, as nations urgently depended on them to access analysis pipelines and efficiently manage data. Currently, large scale European projects, such as the European Genomic Data Infrastructure (GDI) and EOSC-ENTRUST ( Spalding et al., 2023; Sætrom and Negru, 2024), build on these established infrastructures by leveraging their distributed networks of national nodes and central coordination mechanisms to achieve both European-level integration and effective national implementation. In addition, while the bioinformatics ecosystem has increasingly embraced the FAIR (Findable, Accessible, Interoperable, and Reusable) principles for data ( Rocca-Serra et al., 2023; Wilkinson et al., 2016), software ( Barker et al., 2022; Goble et al., 2020; Patel et al., 2023), and training ( Garcia et al., 2020), the effective implementation of these principles remains challenging. The national bioinformatics infrastructures were identified to be necessary mechanisms to overcome these challenges ( Soranzo and Goble, 2023).

A keystone of national bioinformatics infrastructures, the national professional organisations in bioinformatics play roles in networking facilitation, training or job placement. Importantly, through the implicit national representation, these professional organisations can ethically advocate the interests of the profession, broaden participation, drive change, develop and deploy standards and ethical guidelines ( Mourad et al., 2018; Suddaby et al., 2002). Bioinformatics professional organisations exist in most European countries, although their role depends on national circumstances. Guidelines on how to establish bioinformatics communities and scientific professional societies can be found in Budd et al. (2015) and Gaëta et al. (2017).

We present in this paper the priming and evolution of the bioinformatics community and ecosystem in Romania between 2017 and 2024. We describe how the Romanian bioinformatics community evolved into an established national society - the Romanian Society of Bioinformatics (RSBI), the process of preparing a national bioinformatics infrastructure and the steps towards Romania’s ELIXIR Membership.

This paper aims to (1) document the historical activities and the structure of these organizations, providing an institutional record to support continuity, institutional memory, and informed future decision-making; (2) provide policymakers with an overview of academic initiatives in bioinformatics and highlight the community’s longstanding interest in developing such structures; (3) review priorities for the established institutional consortium resulting from RSBI-led efforts, the Romanian Bioinformatics Cluster, to fulfill the role of an ELIXIR Node and provide a documented support for wider discussions on its evolution; and (4) share the experiences from building community-driven scientific structures, including insights from unsuccessful projects.

## Establishment of a professional society in bioinformatics in Romania

The RSBI was established as a bioinformatics professional society following a preparation phase that started in 2017 with a mission to enhance Romania’s bioinformatics capacity. At that time, Romania lacked the critical mass necessary to develop self-sustaining bioinformatics programs and had minimal collaborations among bioinformatics groups across the country.

The development of the bioinformatics community into a national society was closely tied to a series of bioinformatics workshops organised by the founding group. By co-organising workshops in partnership with various host organisations, and establishing a presence in different academic centres (Bucharest, Cluj-Napoca, Iaşi, Timişoara), RSBI achieved widespread national distribution and facilitated connections among scientists from its inception.

RSBI officially came into existence in 2019, following the approval by the Romanian Government for RSBI to adopt the national reference, an approval given after the founding community demonstrated national representation. Importantly, the development of the society and the Bylaws of Association were freely debated among the founding members of the association.

Right from the start, RSBI prioritised transparency, a sense of ethics, and achieving a balanced geographical, academic and gender representation. Of note, the initial development was facilitated by a strong involvement of the Romanian diaspora from the Wellcome Sanger Institute and the European Bioinformatics Institute, UK.

RSBI is governed by a board of up to 6 members. Among these members, one serves as the legal representative. The board is chosen by the General Assembly through a voting process, an anonymous voting procedure being adopted starting April 2024.

The society fosters continuous communication among its members, recognizing it as a fundamental aspect of its operations. This communication is facilitated through an email group as well as monthly meetings. Additionally, RSBI ensures engagement with stakeholders and the public through active management of social media accounts ( RSBI, 2018a, 2018b).

### Core activities of RSBI

**Figure 1.**
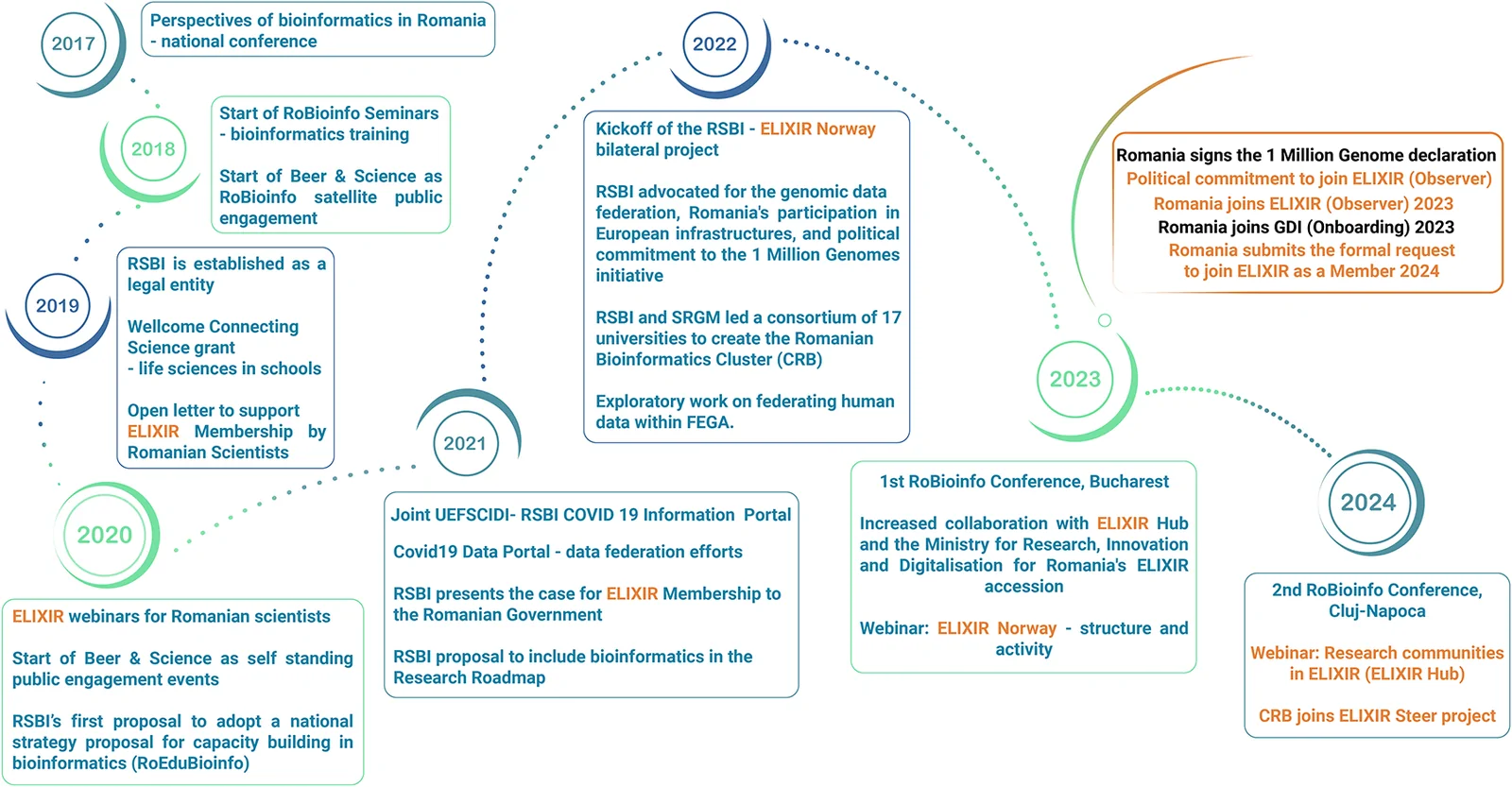
Timeline of RSBI activities: capacity building and progressing toward the ELIXIR ecosystem. Following an initial emphasis on training and public engagement events, RSBI has assumed roles in advocating Romania’s participation in European initiatives and the development of the national bioinformatics infrastructure CRB. The RoBioinfo Seminars started in 2018, and, since then, RSBI organised more than 30 training and 20 public engagement events (Extended data 1). The infrastructure development campaigns included multiple communications campaigns addressed to public administration officials and extensive communication campaigns to the research community. Abbreviations: SRGM - Societatea Română de Genetică Medicală (Romanian Society for Medical Genetics), FEGA - Federated European Genome-phenome Archive, RSBI - Romanian Society of Bioinformatics, UEFISCDI - Romanian Executive Agency for Higher Education, Research, Development and Innovation Funding, CRB - Romanian Bioinformatics Cluster.

RSBI’s initial objectives were to run bioinformatics training at national scale and organise the community so it can provide support to the development of academic bioinformatics training. Although the training events remained the core activity, RSBI developed as a proactive organisation, taking additional roles in building the Romanian bioinformatics ecosystem through engagement with the public and advocacy for infrastructure and policy development ( Figure 1).

Training

The RoBioinfo Seminars, a national bioinformatics training programme (30+ events; Extended data 1), owe much of the initial development to the support provided by groups from EMBL-EBI Training, Ensembl Training, Polish Bioinformatics Society (PTBI), Italian Bioinformatics Society (BITS), and the Hungarian ELIXIR Node. The initial collaborations, coupled with a proactive communication campaign, resulted in an average pre-pandemic participation rate of 20% from neighbouring countries to the RoBioinfo Seminars. In addition to their technical substance, the in-person RoBioinfo Seminars are notable for fostering a scientific and inclusive atmosphere, providing an opportunity for Romanian bioinformaticians to meet and facilitating the development of the national network.

From 2022 to 2024, through bilateral cooperation with ELIXIR Norway, funded by a grant from the Bilateral Fund associated with the EEA and Norwegian Grants 2014-2021, the focus of the workshops expanded to include new topics such as data federation and scientific workflow systems. In 2023, RSBI initiated the annual RoBioinfo Conferences as part of the same project.

Public engagement

Public engagement has been from the very beginning a substantial part of RSBI’s philosophy. Following the model of previously tested initiatives ( Georgescu, 2023), RSBI organised several “Beer and Science” and “Science in schools” events. Initially a satellite event to the RoBioinfo Seminars, “Beer and Science” has since evolved into standalone events. While open to the public, their primary aim remains fostering connections among students, young and established scientists across various disciplines, research groups and scientific interests. We provide in Extended data 2 the RSBI perspective and toolkit for organising Beer and Science events. Importantly, these events had the desired secondary effect of developing a sense of community and social impact within the organisation.

Furthermore, RSBI has also actively contributed to informing the general public on topics of high interest. During the COVID-19 crisis RSBI collaborated with the Romanian Executive Agency for Higher Education, Research, Development and Innovation Funding (UEFISCDI) to better inform the general public ( UEFISCDI, 2020). RSBI also developed information leaflets for public and general medical practitioners on the relevance of federating genomics data (Extended data 3). Importantly, as RSBI is an organisation of scientists, achieving sustained communication with the general public on a large scale can only be accomplished through collaborations with other organisations.

Policy

RSBI has actively contributed to the reform of public policies in genomics and bioinformatics. This involvement includes contributing to the development of national research strategies in life sciences and digitalization, as well as advocating for participation in European initiatives on genomics and bioinformatics. Following an organic approach, RSBI has worked to raise awareness and gather feedback within the scientific community. Subsequently, this feedback has been passed to policymakers, aiming to ensure that perspectives from relevant stakeholders are considered in the policymaking process.

For this purpose RSBI co-organised a series of webinars targeting the research community ( ELIXIR, 2024, 2020) and has disseminated information to the public through press releases, radio interventions and social media, (e.g. ( RSBI, 2023)).

RSBI then engaged with the Romanian Ministry of Research, Innovation and Digitalization (MCID) on matters concerning human genomics and bioinformatics in general. Recognizing the importance of grounding political actions in research realities, RSBI documented and presented to political representatives the scientific motivation for national level approaches to genomics, the opportunities provided by the European bioinformatics infrastructure, and drawbacks of non-participation in such initiatives.

Building upon the groundwork laid by RSBI between 2020 and 2023, and with proactive involvement from the University of Medicine and Pharmacy “Carol Davila”, Bucharest, MCID made the decision in 2023 to sign the ‘1+ Million Genomes’ (1+MG) European initiative ( Romanian Government, 2023) and join the European bioinformatics infrastructure - ELIXIR as Observer ( ELIXIR, 2023).

Attempts towards data federation

Efforts towards data federation in Romania, led by RSBI, did not yield similar levels of success as of above-mentioned initiatives (Extended data 4). While there is recognition of the importance of integrating individual contributions into major European data initiatives like the COVID-19 Data Portal ( Harrison et al., 2021) or the Federated European Genome-phenome Archive (FEGA) ( Freeberg et al., 2021), insufficient resources and incentives hindered active participation and contribution from individual groups.

Despite their slow development, adopting data federation in some form, and establishing the necessary data structures are crucial for numerous research projects. This underscores the significance of having national bioinformatics infrastructures capable of implementing data solutions on a national level and providing support to research groups.

### Conclusions on the establishment of the national professional society

The principles described in ( Budd et al., 2015) and ( Gaëta et al., 2017) proved to be important for the development of RSBI. The clear mission, the commitment to inclusivity, a positive work environment, and the recognition of contributions attracted a substantial number of volunteer contributors. The organisation has evolved based on feedback from its members. For instance, suggestions regarding the need for consistent communication were implemented, resulting in the establishment of monthly RSBI meetings. Of note, sustaining and developing a professional society requires significant community management effort, an activity that usually goes beyond expected voluntary academic involvement. As also mentioned in ( Budd et al., 2015), appropriate compensation for community managers would be a mechanism to ensure sustainability.

Importantly, given the ongoing development of the field and the ubiquitous large national genomics programs, a national bioinformatics society can have a significant impact. To maximise this impact, we add to the previous guidelines the recommendation of 1) establishing working relationships with the public administration and 2) identifying the overlaps and synergies with the bioinformatics infrastructures, e.g. with the National ELIXIR Nodes.

## Towards the national bioinformatics infrastructure - the Romanian Bioinformatics Cluster

Recognizing the deficiencies in both data and training infrastructure, RSBI suggested to the national community the development of an entity capable of conducting nationwide activities to address these challenges.

**Figure 2.**
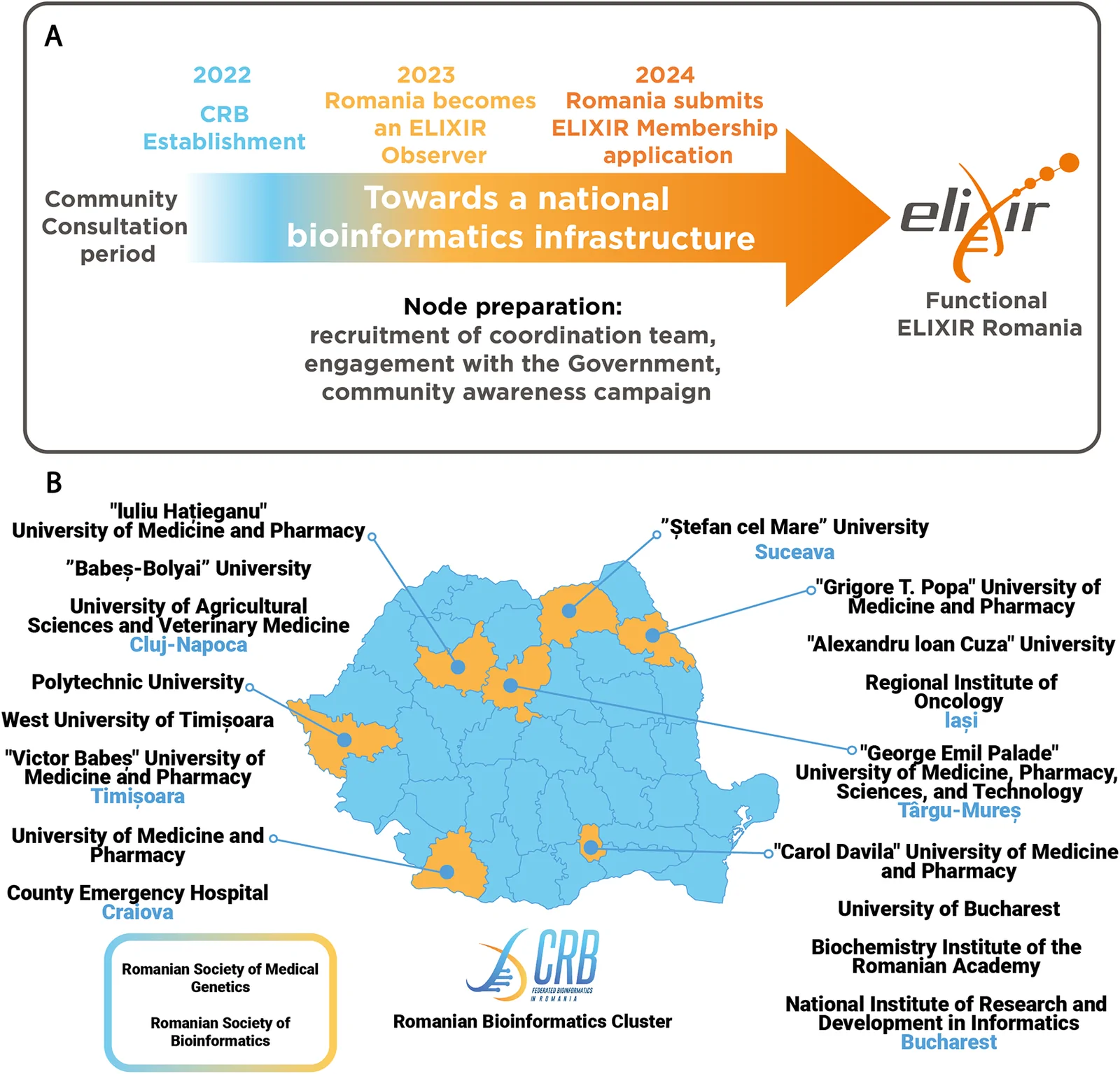
Development and structure of CRB. A) Steps followed in creating a functional ELIXIR Node, starting with community consultation in 2022 the governmental decision to submit the membership request in April 2024 and culminating with Romania's application being approved by the ELIXIR Board in June 2024. The diagram highlights the critical importance of three aspects for a functional ELIXIR Node: community involvement, recruitment of coordinators for data, tools, and training, and engagement with the government. B) Distribution of CRB founding members across Romania: many of the major Romanian research centres and the two professional societies are represented in CRB.

Following a thorough consultation period within the research community, the Romanian Bioinformatics Cluster, or “Clusterul Român de Bioinformatică” in Romanian, hereon CRB, was established in 2022 by seventeen universities and research and medical organisations in collaboration with two national professional organisations ( Figure 2; https://bioinfocluster.ro/). CRB operates as a not-for-profit entity with legal status and a federated structure. Its organisational model draws inspiration from national ELIXIR consortia.

CRB is governed by the General Assembly of Members, in which each member institution has a representative and an executive body, the Board of Directors. The Board of Directors is elected for a 2-year mandate, an anonymous nomination and voting procedure being implemented starting May 2024. The daily administration is carried out by an Administrative Entity, currently “G. E. Palade” University of Medicine, Pharmacy, Science and Technology of Târgu Mureş.

The primary objectives of CRB include:

• **A framework for the development of national bioinformatics training and training resources**. This entails backing programs conducted by RSBI, as well as contributing to the development and upkeep of bioinformatics training resources, and facilitating the training delivery. In practice, within the framework developed by the ELIXIR Training Platform, this will consist in the development of an eLearning ecosystem, supporting the implementation of the FAIR-ification ( Garcia et al., 2020) of training materials, and supporting the train-the-trainer programmes in bioinformatics.
• **Increase the value of data through the development of data infrastructures**. CRB aims to facilitate the development of data infrastructures for molecular data by supporting research organisations in deploying national instances of data structures (eg. portals for pathogen data ( Harrison et al., 2021) or human data ( Freeberg et al., 2021)), implementing novel techniques to data sharing ( Amid et al., 2019), developing data stewardship expertise and ensuring uniform access, e.g. by building on the LS Login authentication service from EOSC-Life .
• **Ease the choice and use of bioinformatics tools**. CRB will aim to achieve this by implementing a distributed help desk system and by supporting the development of a national bioinformatics software ecosystem integrated in the European framework. This will build on advancements in scientific workflow managers ( Crusoe et al., 2022; Di Tommaso et al., 2017; Goble et al., 2021; The Galaxy Community, 2022), software benchmarking ( Pico et al., 2022) and European projects aimed at developing an integrated ecosystem, e.g. https://research-software-ecosystem.github.io/. The support will include contributions to European initiatives, engaging the national community to adopt these advancements, and launching or supporting relevant national instances.

To support these objectives, CRB has developed the Catalogue of Romanian Bioinformatics Services (CaRoBioS; https://bioinfocluster.ro/services/carobios), a peer-reviewed repository of bioinformatics assets for which Romanian groups are the primary developers or maintainers. CaRoBioS aims to improve the discoverability of these assets and to provide constructive feedback to support their development. It also offers a framework to help funders identify initiatives relevant to the national community. Relying on review criteria inspired by the FAIR principles, CaRoBioS helps the community adopt and adapt these principles in practice.

Thanks to its federated structure and strong interaction with ELIXIR, CRB possesses the necessary mechanisms to assist member universities and other national research centres in adopting best practices and adhering to standards. This is particularly significant given that new projects in life sciences are slated for funding in Romania in 2024, and Romania expanded its participation in European research infrastructures through the development of National Nodes for ERIC-MIRRI (Observership; 2023) and Genomic Data Infrastructure (Onboarding; 2024). Harmonising the bioinformatics outcomes and addressing the technical and differing issues to achieve functional interoperability requires inter-institutional approaches, such as those enabled through the CRB structure.

## Discussions - an evolving bioinformatics ecosystem in Romania

The Romanian bioinformatics community has significant growth potential. Based on membership and participation in RSBI-organised events, we estimate that, at the time of publication, the Romanian bioinformatics community is on the order of 200 individuals. This includes fewer than 50 senior core bioinformaticians, alongside a broader group of researchers with strong engagement in bioinformatics across several domains, including computational structural biology, microorganism and human ’omics. This back-of-the-envelope estimate indicates substantial potential for growth, particularly in training, the development of formal academic programmes, and community building.

The initial phase of RSBI’s development was driven by a pressing need to start bioinformatics training, foster a sense of community, advocate for the importance of this profession, and contribute to the establishment of a country-wide network of bioinformaticians.

The present and forthcoming period for the Romanian life sciences is marked by the implementation or planning of several large-scale projects requiring bioinformatics, and Romania’s integration into the broader European bioinformatics ecosystem. Coinciding with these developments, RSBI approached a maturity stage, a stage in which its role as a professional organisation is evolving.

Maintaining a strong sense of community remains RSBI’s fundamental mission. This spirit of community is crucial not only for facilitating training and networking but also for spearheading the adoption of new methodologies. Monthly meetings, annual conferences, training and public engagement events have been and will continue to be effective mechanisms for connecting the community. Alongside these, the establishment of national communities of practice embedded in the ELIXIR communities is anticipated to accelerate the development of the national bioinformatics capacity. As a transversal organisation, RSBI can act as a unifying force for stakeholders, bringing together diverse parties to facilitate discussions in the best interest of national bioinformatics. RSBI can also play a significant role in strengthening the local community by sustainably connecting Romanian bioinformatics professionals in the diaspora with scientists in the country.

The training activities are poised to evolve with the adoption of FAIR principles. As high-quality resources become more available, especially through ELIXIR, there is a need to adjust the training model accordingly. The national bioinformatics training program must transition to a more integrated approach, aligning closely with the broader European resources offered by ELIXIR Nodes.

The engagement with the wider life science community and the public would need to be scaled up. Of note, community engagement and involvement is becoming a prerequisite for major research projects. National professional societies and infrastructures offer the optimal framework for fostering this involvement. RSBI can achieve sustained communication with the general public on a large scale through collaborations with other organisations.

The evolution of bioinformatics as a profession, along with its expanding roles in science and industry, will inevitably raise new ethical considerations. RSBI must gradually transition into assuming the role of an ethics body to address these emerging ethical challenges.

Importantly, RSBI’s activities must be closely linked with those of CRB. Firstly, the infrastructure developed by CRB, and notably the mechanisms to implement FAIR, can be impactful only if adopted by the community. RSBI has the scale and mechanisms to advocate for these principles within the community. Secondly, an ELIXIR Node has the institutional mechanisms to leverage the resources required by RSBI to organise training and public engagement programmes. Finally, the relationship between these two organisations, both developed on ethical principles, offers a framework to maintain checks and balances, ensuring equitable access to resources and appropriate representation of community interests. Although the scope of an ELIXIR Node, which focuses on infrastructure development, differs from that of a national professional organisation, which aims to connect, facilitate career development, and represent the professional community, these entities must work closely, optimise synergies, and manage overlaps.

## Author contributions

BM developed the concept with input from HLB, MM and MA. BM performed the research and prepared the manuscript. MG, DM and MDL prepared the figures included in the manuscript. CZB prepared the Supplementary Material 2. MM, MG, MA, HLB and CZB contributed to the preparation and revision of the manuscript.

## Data Availability

No data are associated with this article. Zenodo: Extended Data 1: The list of RoBioinfo Seminars, workshops and webinars organised between November 2017 and April 2024. https://doi.org/10.5281/zenodo.13235287 ( RSBI, 2024a). This project contains the following extended data:
- The RoBioinfo Seminars, workshops and webinars.pdf Zenodo: Extended Data 2: Beer and Science - Public Engagement Toolkit. https://doi.org/10.5281/zenodo.13235179 ( RSBI, 2024b). This project contains the following extended data:
- RSBI Beer and Science toolkit.pdf Zenodo: Extended Data 3: Human Genomics - Public Engagement Leaflets - Romanian. https://doi.org/10.5281/zenodo.13150360 ( RSBI, 2024c). This project contains the following extended data in Romanian with corresponding ENglish translations: translations:
- RSBI Public Engagement leaflet - medical doctors.pdf - RSBI Public Engagement leaflet - public.pdf Zenodo: Extended Data 4: Data federation advocacy attempts. https://doi.org/10.5281/zenodo.13235768 ( RSBI, 2024d) This project contains the following extended data:
- Data federation advocacy attempts.pdf Data are available under the terms of the Creative Commons Attribution 4.0 International license (CC-BY 4.0).
