# Supplementary material for: From the establishment of a national bioinformatics society to the development of a national bioinformatics infrastructure
Source: F1000Res. 2026 Jun 17;13:1002. Originally published 2024 Sep 3. [Version 2] doi: 10.12688/f1000research.153895.2 (PMC13550889; doi:10.12688/f1000research.153895.2)
Supplement: Peer review history

# Reviewer report: Reviewer response for version 2

**DOI:** 10.5256/f1000research.195294.r494496

**Published:** 2026-07-09

**Authors:** Carol Goble

**Affiliations:** 1 The University of Manchester, Manchester, England, UK

The authors have made many of the changes requested in my previous review, notably an explanatory piece on the objective of the paper and the inclusion of an initial version of a Catalogue of Romanian Bioinformatics Services and the procedures for being listed therein. 

 The article aim is chiefly to document the history of establishing the RSBI society and then the Romanian Bioinformatics Cluster. This it does well. It lays out more clearly the primary objectives of the CRB (training, access to data and tools) and the scale of the bioinformatics community. It emphasises challenges of building a community and the importance of public engagement.

 It is interesting to see that federation proved to be difficult to establish and it would have been good to have delved into the reasons a bit further. 

 There are now hints towards the areas of need/strength (computational structural biology, microorganisms, human omics) but these are hints, and the catalogue doesn't yet yield much insight into the biological areas of strength and need for the CRB. It is, however, an encouraging initiative to have a catalogue established.

 What is still confusing is that the paper starts strongly with a statement about ELIXIR and wishing to establish a Romanian Node, but then back peddles on reviewer comments about contributions ELIXIR would make to the Romanian Node and vice versa, citing that this cannot be done until the Node is established. On the contrary, in this opinion piece the case could be made and strengthened. 

 While it is understood that comments on figures can be ignored, it is disappointing, given that more than one reviewer made the same suggestion on the map figure. 

 In summary, the article is clearer and improved and will contribute to the community a document recording the progression of the Romanian bioinformatics community. It does feel like a missed opportunity to make more specific the opportunities of Romania of joining ELIXIR and ELIXIR gaining Romania, strengthening the case.

 Minor point - Page 5 "European Bioinformatics Institute, UK" gives the impression that the EBI is a UK institute. It is not. It is a German institute (the EMBL-EBI Outpost) hosted in the UK.

Is the topic of the opinion article discussed accurately in the context of the current literature?

Yes

Are arguments sufficiently supported by evidence from the published literature?

Yes

Are all factual statements correct and adequately supported by citations?

Yes

Are the conclusions drawn balanced and justified on the basis of the presented arguments?

Partly

Reviewer Expertise:

Joint Head of Node ELIXIR-UK, ex-member of ELIXIR Interim Board, ExCo ELIXIR Interoperability Platform, areas: Digital Research Infrastructure, FAIR and Open Science, workflows, metadata. Lead of several ELIXIR flagship services.

I confirm that I have read this submission and believe that I have an appropriate level of expertise to confirm that it is of an acceptable scientific standard.

# Reviewer report: Reviewer response for version 1

**DOI:** 10.5256/f1000research.168851.r321799

**Published:** 2024-10-12

**Authors:** Carol Goble

**Affiliations:** 1 The University of Manchester, Manchester, England, UK

The paper is two parts, both concerning the establishment of a bioinformatics national community in Romania. The first part describes how and why a professional society was established; the second the steps towards a national node of the ELIXIR research infrastructure.

 The article is informative and quite well written and describes the high level vision and the steps taken.

 As an opinion piece it is short on opinion. It is unclear if the objective is to provide an announcement of the establishment of a society and an ELIXIR Node, or if there is some more significant discussion regarding the specific challenges within Romania or lessons that other countries might learn.

- Some clarity is needed in what is meant by a “national bioinformatics infrastructure”, which in this case is an ELIXIR Node. What an ELIXIR Node is, and the many forms of Nodes (there are 25 nodes including Romania) needs to be better described – most readers will not know. The term “infrastructure is open to interpretation
- In what way does the RSBI differ from other National societies? What did you find useful and less useful from using the guidelines you cite?
- What are the unique or special offerings of the Romanian Node to ELIXIR and what does Romania need for its bioinformatics community? There are 30+ communities and focus groups in ELIXIR – which ones are strengths or beneficial growth areas for Romania? The objectives of the CRB are rather high level and non-national specific. Does Romania have strengths in areas of rare disease, precision medicine, agritech, structiural bioinformatics, e-learning, AI etc
- What is the scale of a country-wide network of bioinformaticians?  How many people? Data services? There are 17 organisations – are these funded as a network like ELIXIR UK or as a funded programme of joint work like ELIXIR-CZ? Is the CRB supported by a national award or subscriptions or in kind?
- Data federation takes many forms and is complex (it is not just FEGA) – two paragraphs are unclear what federation was expected or tried.
- A national ELIXIR Node is expected to interact with other nodes. Training with Hungary and Poland was mentioned and participation in GDI. Are here other opportunities given your CRB objectives?

In summary the paper would benefit from a clearer statement of its message and a clearer statement on the national needs and national resources.  It can also provide useful critical insights on the challenges and resources needed to establish a national infrastructure useful for other nations.

 Minor points

 Introduction

- The introduction’s history of ELIXIR should add that it was founded in 2013. It isn’t clear what is meant by “second phase” and I suggest you drop this phrase.
- The projects GDI, EOSC-ENTRUST should be cited. The sentence that these projects “reply on these structures for their European and national reach” is unclear. Do you mean they rely on national nodes? It is not clear who “these structures” and “they” refers to. 
- National infrastructures were identified as “adequate” mechanisms is a strange statement. Needed or necessary or just mechanisms, perhaps.
- Figure 1 is useful but too busy to read
- Figure 2 A is unnecessary

Is the topic of the opinion article discussed accurately in the context of the current literature?

Yes

Are arguments sufficiently supported by evidence from the published literature?

Yes

Are all factual statements correct and adequately supported by citations?

Yes

Are the conclusions drawn balanced and justified on the basis of the presented arguments?

Partly

Reviewer Expertise:

NA

I confirm that I have read this submission and believe that I have an appropriate level of expertise to confirm that it is of an acceptable scientific standard, however I have significant reservations, as outlined above.

# Response

**Published:** 2026-01-15

**Authors:** Bogdan Mirauta

**Affiliations:** Romanian Bioinformatics Cluster, Targu Mures, Romania

We thank the reviewer for the detailed and constructive feedback. We delayed submitting our responses in the expectation that the process of ELIXIR membership for Romania, one of the important outcomes documented in this article, would be finalized; however, the Romanian Parliament’s decision required under Romanian law has taken longer than anticipated and is currently pending in February  2026.

 While this extended the response timeline, it allowed us to address an important request from the reviewers. Following the reviewers’ suggestions to address the bioinformatics resources in Romania, we have launched the initial version of the Catalogue of Romanian Bioinformatics Services (CaRoBioS; https://bioinfocluster.ro/services/carobios), in which the first resources have recently undergone peer review. This live, peer-reviewed registry will contribute to a reliable description of the bioinformatics portfolio and help identify the specific contributions of Romanian bioinformatics to ELIXIR.

 In addition, we have clarified the scope of the paper by adding a dedicated paragraph at the end of the Introduction. While one objective of this article is to facilitate the establishment of the Romanian ELIXIR Node (CRB), we note that its main purpose is to document the historical activities of the professional organization Romanian Society of Bioinformatics (RSBI), providing a record that is important for understanding the community’s longstanding engagement, guiding future initiatives, and informing policymakers and funders about the evolution of bioinformatics in Romania. Additionally, we clarified that the Romanian ELIXIR Node is forthcoming and has not yet been established.

**Additional major comments**

 We thank the reviewer for highlighting the need to clarify the Romania’s role in ELIXIR. We have added a paragraph addressing some of the objectives for the Romanian Node but did not address its contribution in the wider ELIXIR network. We note that, as the ELIXIR membership process is not yet finalized, we cannot yet accurately address points related to Romania’s contributions within ELIXIR. However, we have developed CaRoBios, a registry of resources, that will likely help identifying resources that can benefit the wider network. We note that one of the criteria used in evaluating these resources is their potential within  ELIXIR.

 Similarly, to address the question “what is meant by a national bioinformatics infrastructure”, an unequivocal definition will be provided once the ELIXIR membership process is finalized. However, RSBI made significant steps in defining this infrastructure by establishing its administrative structure, i.e CRB, a consortium with legal status, and identifying the priorities in terms of data, tools and training. 

 We also wish to clarify that, at present, neither CRB nor RSBI receives dedicated core funding. CRB’s future funding opportunities are largely contingent on the Parliament’s approval of ELIXIR membership, while RSBI is primarily funded through member fees. 

 At the beginning of the Discussion section, we added information on our estimate of the size of the bioinformatics-dedicated community in Romania. Although this estimate is approximate, it provides readers with a clearer understanding of the community’s current scale and its potential for future development.

 Regarding potential interactions between CRB (as the main candidate for Romania’s ELIXIR Node) and other ELIXIR Nodes, these are highly dependent on the completion of the ELIXIR membership process for Romania. Institutional interactions are expected primarily at the CRB level rather than at the level of the professional society. We would also like to emphasize that this paper focuses on the role of professional society RSBI in developing the national bioinformatics ecosystem; therefore, future interactions at the CRB level are beyond the scope of this manuscript. 

 Regarding data federation, we acknowledge that it is a complex concept with multiple possible implementations and that it offers clear benefits across many domains beyond the well-established human ’omics–related use cases. While valuable lessons were learned from the exploratory efforts described in this paper, we are unable to provide further details on this topic at this stage.

 We agree with the reviewer on the importance of highlighting the most relevant elements of the cited guidelines and direct the reviewer to the discussion in the first paragraph of the section “Conclusions on the Establishment of the National Professional Society”.

**Minor comments**

 We have addressed the minor points raised by the reviewer by adding the ELIXIR foundation year, clarifying the phrasing regarding how GDI and EOSC‑ENTRUST leverage established pan-European infrastructures, including references to these projects, simplifying the contents of Figure 1 and implementing other minor revisions.

# Reviewer report: Reviewer response for version 1

**DOI:** 10.5256/f1000research.168851.r321795

**Published:** 2024-09-30

**Authors:** Federico Zambelli

**Affiliations:** 1 University of Milan, Milan, Italy

The authors outline the establishment of the Romanian Society of Bioinformatics and the efforts towards building a national biological data infrastructure, including the formation of a tentative ELIXIR Node. 

 Many of the problems and hurdles in the process are common with other countries, including the difficulty of interacting with policymakers and decision-making bodies to establish clear road maps, which are critical components in the construction and maintenance of any infrastructure, including those dedicated to scientific data.

 The manuscript does a good job of describing Romanian bioinformatics' current situation and ambition, and the initiative's promoters are to be commended for their efforts in integrating national efforts in the European context. However, a more in-depth description of the current bioinformatics portfolio of resources, services, industry interactions (if any), interests, main actors, and expertise in Romania would help the reader understand the sector's growth potential in the country.

Is the topic of the opinion article discussed accurately in the context of the current literature?

Yes

Are arguments sufficiently supported by evidence from the published literature?

Yes

Are all factual statements correct and adequately supported by citations?

Yes

Are the conclusions drawn balanced and justified on the basis of the presented arguments?

Yes

Reviewer Expertise:

Bioinformatics, transcriptomics, epigenomics, research infrastructures, cloud computing.

I confirm that I have read this submission and believe that I have an appropriate level of expertise to confirm that it is of an acceptable scientific standard, however I have significant reservations, as outlined above.

# Response

**Published:** 2026-01-15

**Authors:** Bogdan Mirauta

**Affiliations:** Romanian Bioinformatics Cluster, Targu Mures, Romania

We thank the reviewer for their insightful comments and for recognizing the challenges faced in establishing a national bioinformatics infrastructure, including interactions with policymakers and decision-making bodies.

 We delayed submitting our responses in the expectation that the process of ELIXIR membership for Romania, one of the important outcomes documented in this article, would be finalized; however, the Romanian Parliament’s decision required under Romanian law has taken longer than anticipated and is currently pending in February 2026.

 While this extended the response timeline, it allowed us to address an important request from the reviewers. Following the reviewer's suggestion to address the portfolio of bioinformatics resources in Romania, we have launched the initial version of the Catalogue of Romanian Bioinformatics Services (CaRiBioS; https://bioinfocluster.ro/services/carobios), in which the first resources have recently undergone peer review. This live, peer-reviewed registry will contribute to a reliable description of the bioinformatics portfolio and help identify the specific contributions of Romanian bioinformatics to ELIXIR. We note that, although rigorously developed, the catalogue is still in its early stages, with the first evaluated resources added in December 2025, and therefore it does not yet provide an exhaustive list of Romanian bioinformatics resources. We have added a paragraph describing CaRoBioS in the manuscript.

# Reviewer report: Reviewer response for version 1

**DOI:** 10.5256/f1000research.168851.r321797

**Published:** 2024-09-25

**Authors:** Francis Ouellette

**Affiliations:** 1 Bioinformatics, Montreal, Canada

The authors of the opinion article "From the Establishment of a National Bioinformatics Society to the Development of a National Bioinformatics Infrastructure" present the evolution of a bioinformatics national community. They describe the existing bioinformatics infrastructure that the Romanian national bioinformatics community will use. This opinion is part of their process to establish and evolve the Romanian Node into a full member of  Elixir Europe. 

 This opinion piece should expand on and clarify a few points. These comments are made in light of the nature of this submission: it is the expression of the author's opinion, not mine. 

 1. This article is well-written, but the underlying objectives need to be clarified. This position paper would be more explicit to the reader if the authors expressed their intent to develop a national bioinformatics infrastructure and why they are doing what they describe.

 2. Knowing the reading audience is essential to consider when writing this opinion. If the audience is complex and diverse (e.g., politicians, senior biologists, new computer scientists, and trainees), you may need to target some sections for each audience.  

 3. Another important point:

 Are there particular biological questions that need to be answered by this community?

 Do they need trained bioinformaticians, dedicated resources, cloud computing, specific tools, or specialized databases to answer these questions?

 Is there a need for the medical community to be able to interpret human genomes?

 Specific environmental microbiomes?

 Do veterinarians need to understand and deal with specific outbreaks?

 Do farmers have specific pest management challenges resulting from global warming they have never anticipated?

 Are the current biology and health sciences programs ill-equipped to address these challenges?

 If so, the community may need to train bioinformatics people at all levels (undergraduates, graduate, post-doc, and professional development programs), and for that work to get done, you will also need funding (scale of funding necessary). An opinion piece like this one is an opportunity to explain what the needs are, how to achieve these needs and defend the budget necessary to make this possible. 

**Comments about the figures:**

 4. Figure 1: The idea of a timeline is excellent. This timeline represents many of the items detailed in the text. The figure has too much text and should be redone with one or two points for each critical time point. Can a reference tag be used in the text? I am unsure about the rules for this journal, but it might be useful to consider a separate table with all the acronyms used in this opinion.  

 5. Figure 2 A: This figure (2A) is not helpful here. It repeats much of the text in the opinion piece.

 6. Figure 2 B: Maps are great information tools, but could this information also be useful in a table?

 1- City

 2- Name of university/Institution/company (with URL)

 3- Department(s) involved

 4- Bioinformatics expertise (maybe develop 5 or 6 codes for different disciplines? 

     Medical genetics, Computer Assisted Drug 

     development, HPC, Database or Algorithm development or any other 

     specialities. 

 5- Human-centric? Plant? Vetenary? Prokaryotic? Viruses? Ecology? Medicine?

 6- Number of scientists involved in this community (group leader/trainees?) at this institution.

 Then, on this map, you could mark the cities (with orange counties where such institutions reside, as you have it now), and with this table, it would be a good community status at this point. Over the years, you could show a growing orange wave across Romania!

Is the topic of the opinion article discussed accurately in the context of the current literature?

Yes

Are arguments sufficiently supported by evidence from the published literature?

Yes

Are all factual statements correct and adequately supported by citations?

Yes

Are the conclusions drawn balanced and justified on the basis of the presented arguments?

Partly

Reviewer Expertise:

Computational biologist, member of the Elixir SAB for the last 8 years.

I confirm that I have read this submission and believe that I have an appropriate level of expertise to confirm that it is of an acceptable scientific standard, however I have significant reservations, as outlined above.

# Response

**Published:** 2026-01-15

**Authors:** Bogdan Mirauta

**Affiliations:** Romanian Bioinformatics Cluster, Targu Mures, Romania

We thank the reviewers for their comments. We delayed submitting our responses in the expectation that the process of ELIXIR membership for Romania, one of the important outcomes documented in this article, would be finalized; however, the Romanian Parliament’s decision required under Romanian law has taken longer than anticipated and is currently pending in February  2026.

 While this extended the response timeline, it allowed us to address an important request from the reviewers. Following the reviewers’ suggestions to address the bioinformatics resources in Romania, we have launched the initial version of the Catalogue of Romanian Bioinformatics Services (CaRiBioS; https://bioinfocluster.ro/services/carobios), in which the first resources have recently undergone peer review. This live, peer-reviewed registry will contribute to a reliable description of the bioinformatics portfolio and help identify the specific contributions of Romanian bioinformatics to ELIXIR.

 In addition, we have clarified the scope of the paper by adding a dedicated paragraph at the end of the Introduction. While one objective of this article is to facilitate the establishment of the Romanian ELIXIR Node (CRB), we note that its main purpose is to document the historical activities of the professional organization Romanian Society of Bioinformatics (RSBI), providing a record that is important for understanding the community’s longstanding engagement, guiding future initiatives, and informing policymakers and funders about the evolution of bioinformatics in Romania. Additionally, we clarified that the Romanian ELIXIR Node is forthcoming and has not yet been established.

**Comments 1.1, 1.2**

 We thank the reviewer for their positive feedback and for highlighting this need to clarify the purpose of the paper. To clarify the objectives of the paper, we have added a dedicated paragraph at the end of the Introduction. This paragraph outlines the rationale of the study and implicitly helps clarify the intended readership. 

**Comments 1.3 **We thank the reviewer for raising the question of the specific biological questions and training needs of the community. Although we conducted surveys to identify training requirements, the results did not allow us to define precise domain-specific directions, as responses reflected a highly heterogeneous community, including groups working in human ’omics, plant and animal biology and microorganisms. Nevertheless, we identified several cross-cutting challenges shared across domains, particularly in data management and data access. Accordingly, we have included data stewardship as one of the priorities of the Romanian Cluster of Bioinformatics (CRB) and note that this is also a recognized priority across the wider ELIXIR community. We also point out that the newly launched framework CaRoBioS will result in the medium term in identifying priority areas for Romanian bioinformatics.

**Comments on figures**

 We thank the reviewer for the suggestion to simplify Figure 1. In response, we have revised the figure and included a new version with substantially reduced text to improve clarity and readability.

 We agree that much of the information shown in Figure 2A is also described in the text. However, we believe that this panel provides a concise visual representation of the chronology leading to potential ELIXIR membership, which facilitates improved understanding of the sustained, long-term commitment of the bioinformatics-dedicated community in Romania.

 While we acknowledge the reviewer’s suggestion to present the information shown in Figure 2B in tabular form, we consider that the geographical map more intuitively conveys the national distribution and spatial coverage of CRB member institutions across Romania. We also agree that providing additional details about each institution would be valuable and will consider including these details in a prospective article focused on describing the aims, structure and potential impact of CRB article once the process of Romania’s membership to ELIXIR is completed.
